# m6Acomet: large-scale functional prediction of individual m^6^A RNA methylation sites from an RNA co-methylation network

**DOI:** 10.1186/s12859-019-2840-3

**Published:** 2019-05-02

**Authors:** Xiangyu Wu, Zhen Wei, Kunqi Chen, Qing Zhang, Jionglong Su, Hui Liu, Lin Zhang, Jia Meng

**Affiliations:** 1 Department of Biological Sciences, Xi’an Jiaotong-Liverpool University, Suzhou, 215123 Jiangsu China; 2 Institute of Ageing & Chronic Disease, University of Liverpool, L7 8TX, Liverpool, UK; 3 Institute of Integrative Biology, University of Liverpool, L7 8TX, Liverpool, UK; 4 Present address: Harvard T.H. Chan School of Public Health, Harvard University, 655 Huntington Avenue, Boston, MA 02115 USA; 5 Department of Mathematical Sciences, Xi’an Jiaotong-Liverpool University, Suzhou, 215123 Jiangsu China; 6 School of Information and Control Engineering, China University of Mining and Technology, Xuzhou, Jiangsu China

## Abstract

**Background:**

Over one hundred different types of post-transcriptional RNA modifications have been identified in human. Researchers discovered that RNA modifications can regulate various biological processes, and RNA methylation, especially N6-methyladenosine, has become one of the most researched topics in epigenetics.

**Results:**

To date, the study of epitranscriptome layer gene regulation is mostly focused on the function of mediator proteins of RNA methylation, i.e., the readers, writers and erasers. There is limited investigation of the functional relevance of individual m^6^A RNA methylation site. To address this, we annotated human m^6^A sites in large-scale based on the guilt-by-association principle from an RNA co-methylation network. It is constructed based on public human MeRIP-Seq datasets profiling the m^6^A epitranscriptome under 32 independent experimental conditions. By systematically examining the network characteristics obtained from the RNA methylation profiles, a total of 339,158 putative gene ontology functions associated with 1446 human m^6^A sites were identified. These are biological functions that may be regulated at epitranscriptome layer via reversible m^6^A RNA methylation. The results were further validated on a soft benchmark by comparing to a random predictor.

**Conclusions:**

An online web server m6Acomet was constructed to support direct query for the predicted biological functions of m^6^A sites as well as the sites exhibiting co-methylated patterns at the epitranscriptome layer. The m6Acomet web server is freely available at: www.xjtlu.edu.cn/biologicalsciences/m6acomet.

**Electronic supplementary material:**

The online version of this article (10.1186/s12859-019-2840-3) contains supplementary material, which is available to authorized users.

## Background

N6-methyladenosine (m^6^A) is one of the most common RNA post-transcriptional chemical modifications. It is formed with an addition of a methyl group at the 6′ position of adenosine in RNA [1]. It is abundant in mRNA, snRNA and rRNA among plants, viruses and eukaryotes [2, 3]. In mammals, methyltransferases (m^6^A writer), such as METTL3, METTL14 and WTAP, together with demethylases (m^6^A eraser), and YTH domain family of proteins (m^6^A reader), regulate the complex reverse mechanism of m^6^A [4]. The m^6^A was found to influence diverse biological regulations such as RNA stability [5], heat shock response [6], and circadian clock [7] etc. Diseases, such as cancer [8] are proved to be regulated by m^6^A as well. Current research focuses more on the overall functions or regulations involving m^6^A. However, the biological function of each individual RNA methylation site is not exactly known. Although the regulatory roles of several specific methylation sites have been elucidated, it is very expensive to identify the functions of RNA methylation sites with wet-lab experiments. Instead, computational approach may provide a viable venue. It is possible that the functions of each individual RNA methylation site can be predicted from the statistical evidence such as strong correlation with the expression level of genes whose functions are already known.

The regulatory functions of methylation sites in biological processes are still under research [9–11]. It is conceivable to assume that m^6^A sites that have similar properties would share similar biological functions. Indeed, our previous studies showed that the RNA methylation sites consisting of an epitranscriptome module, which is a number of RNA methylation sites whose methylation level are co-regulated across different experimental conditions, are more likely to be functionally enriched compared to a random module [12, 13]. This strongly suggests that the epitranscriptome functions of the RNA methylation sites may be identified based on existing high-throughput sequencing data. It is meaningful to investigate the regulatory role of these sites by constructing the co-methylation network with the guilt-by-association principle. The guilt-by-association is a validated principle in network research, which states that if two patterns share some similar properties, they are most likely to share a connection. To be more specific, gene pairs are more likely to be functionally related if they show similar expression patterns across samples [14]. This principle has been widely applied in lncRNA functional prediction by the protein-protein interaction network [15], co-transcription factor network, and co-expression network [14]. In our research, we suppose that, if both methylation sites are hyper- or hypo-methylated simultaneously across various samples, they will be considered co-methylated and often of related biological interests. In the co-methylation network, each node represents a methylation site, and each edge denotes a strong correlation or anti-correlation between each pair of sites.

The datasets used for generating the methylation level on sites in this program are all produced by the MeRIP-Seq technique [1, 16]. Methylated RNA immunoprecipitation sequencing (MeRIP-Seq) technique was developed to investigate m^6^A in epitranscriptome analysis [17]. The mRNAs which contain m^6^A sites are first fragmented into short pieces of ~ 100 bp long, following which fragments with methylation sites are filtered by antibodies in immunoprecipitate as IP samples, while raw fragments are treated as Input control samples [18]. After mapping both the reads of eluted IP sample and control (Input) sample back to the reference genome, the peak-calling or methylation evaluation algorithm will be employed to detect the m^6^A peaks for furthering investigation [19]. The mi-CLIP [20] and the m^6^A-CLIP [21] were developed recently to generate single-base resolution m^6^A profile, and the upcoming data sets were utilized to obtain the m^6^A sites directly in the project. The principle of mi-CLIP is to bind the cross-linking RNA-m^6^A antibody to specific sites where mutagenesis will occur during reverse transcription of the antibody-bound RNA. Trucations or C-T transitions, which are mutagenesis signatures, can be sequenced to precisely map m^6^A sites. The m^6^A-CLIP located thousands of m^6^A residues using cross-linking immunoprecipitation technique (UV CLIP) with high accuracy since only the m^6^A-containing oligonucleotide can attract the m^6^A antibody.

Before constructing the co-methylation network, the matrix which gives the methylation level on each site over various samples needs to be constructed. However, preprocessing is required for the raw data due to technical or biological biases. The DESeq2 [22] is a R package which uses shrinkage estimation for fold changes, and dispersion for gene-level differential analysis with RNA-Seq data. The reproducibility and stability of results are improved by shrinkage estimators after using DESeq2. This algorithm can reduce type-I errors and offer consistent performance on small studies. Guanine-cytosine (GC) content is one of the critical technical variabilities. It was shown to have significant impact on m^6^A-seq [23] and other sequencing techniques such as RNA-seq and ChIP-seq. The CQN algorithm developed by Hansen [24] is aimed to reduce systematic bias in GC content. It combines the robust generalized regression and conditional quantile normalization to improve the precision of gene expression level measurement. In our project, DESeq2 and CQN are applied to estimate the methylation level of each m^6^A site.

After building the complex network, cellular modules were identified for further annotation with gene ontology (GO). The GO is a bioinformatics initiation to unify gene product within species [25]. We mainly used GO for annotating enrichment analysis on gene sets to describe the functions of a specific gene list. The GO enrichment analysis will determine which GO terms are represented, generate the GO term list with statistical evidence such as the *p*-value. The GO terms may be classified into three main categories: biological process (BP), cellular component (CC) and molecular function (MF). To improve the annotation performance, the enriched GO terms will be reduced to generic GO slim terms, by skipping specific fine-grained terms, which is useful when board classifications of function annotation are required [26].

In this project, we computationally predicted the biological functions that are likely to be associated with individual m^6^A RNA methylation sites. Using bioinformatics methods such as clustering, network topological analysis, as well as enrichment analysis. The results may be queried directly on a public webserver, which provides predicted functions for each individual RNA methylation site.

## Results

### Selection of raw m^6^A sites and normalization

**Fig. 1:**
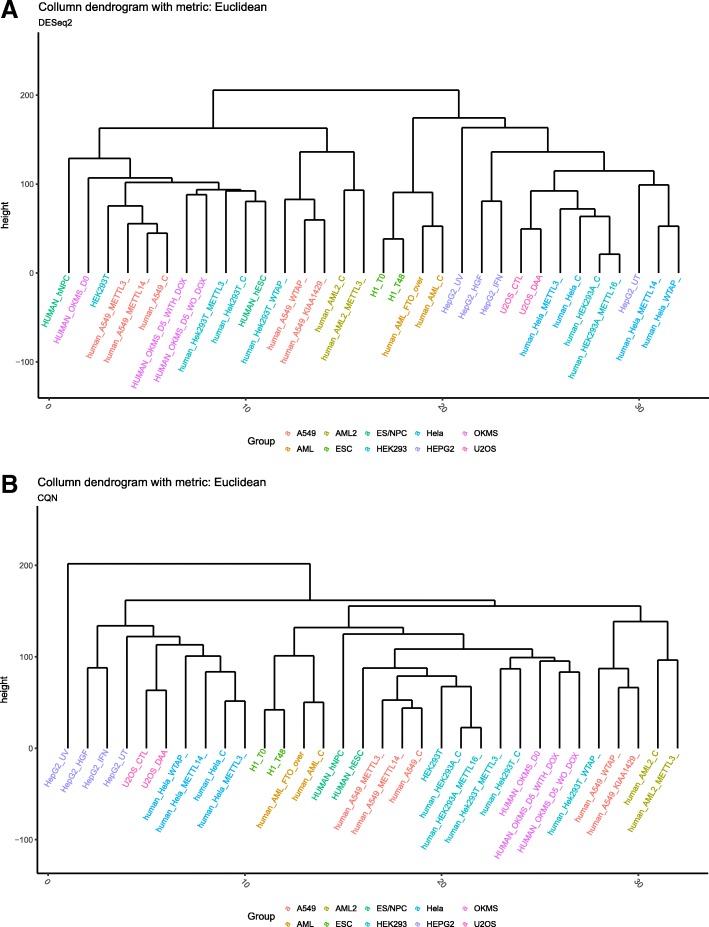
Correction of technical variability. **a** The clustered dendrogram of samples before applying CQN to remove technical variability. Many highly related samples are not clustered closely. **b** The clustered dendrogram of samples after applying CQN to remove technical variability. More related samples are clustered together, suggesting that the application of CQN in the analysis is very effective

After filtering the methylation sites corresponding to lowly expressed genes with low gene expression level and low read count quantity in IP and Input samples, the raw predicted single-base resolution human m^6^A sites were reduced from 69,446 to 36,542. Furthermore, 17,758 sites were discarded to reduce the number of neighboring sites corresponding to the same gene that are very close to each other. A total of 13,415 sites with relatively higher median absolute deviation (over 0.4) among the remained 18,784 sites were kept. We believe that the m^6^A sites remained after selection should be statistically significant. After merging the biological replicates from the same condition together with methylation level estimation by DESeq2, the GC content bias were normalized by CQN. The dendrograms in Fig. 1, constructed using Euclidean distance as the metric, helped us insight into the joint distribution between samples with and without the CQN normalization. Samples from the same cell line and experiment were labelled with the same color. Samples of the same color were not clustered together in the dendrogram without CQN (Fig. 1a). In contrast, after the CQN normalization, almost all the conditions from the same cell line were clustered into the same group with highly correlated methylation patterns (Fig. 1b). This indicates that the GC content biases were removed. Additionally, we tested the relative importance of each individual samples, and found no outliers (Additional file 1: Figure S1). besides, although the samples perturbed with m^6^A enzymes represent an abnormal kind of methylation profiles and may induce more bias, we didn’t observe significant difference between them in determining the topology of the co-methylation network (Additional file 1: Figure S2).

### Co-methylation network construction

**Fig. 2:**
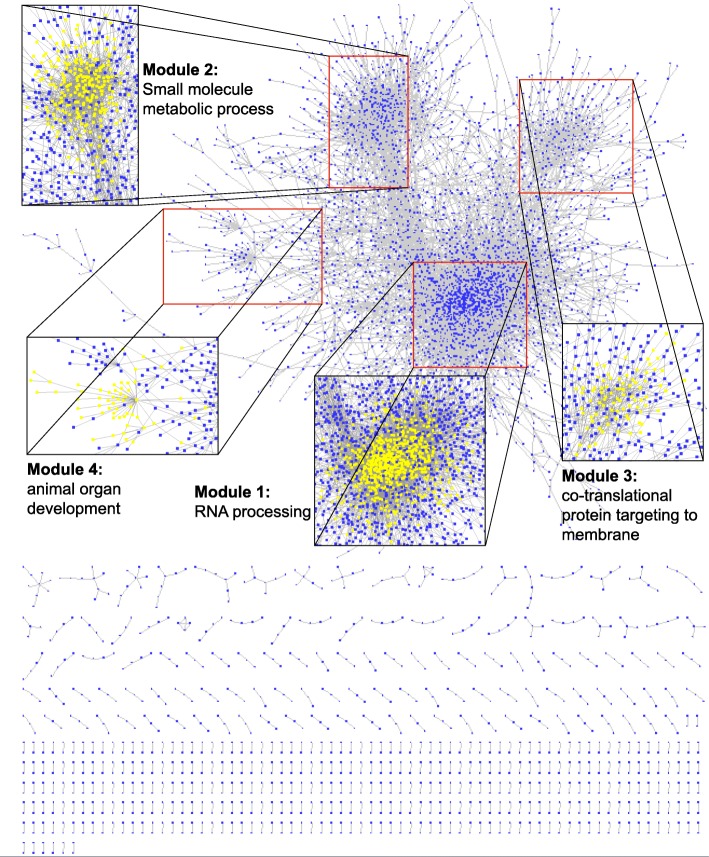
Visualization of the co-methylation network in Cytoscape. A total of 18,477 edges and 13,363 nodes make up this co-methylation network. The m6A sites are represented by blue nodes, and gray lines represent the high positive and negative correlation between each node. Majority of the sites (91.5%) were clustered into a huge module, and few sites share high correlation in methylation level within small modules. Four largest modules were amplificated and labelled in yellow, and the most significant Gene Ontology term of each module was labelled as well

**Fig. 3:**
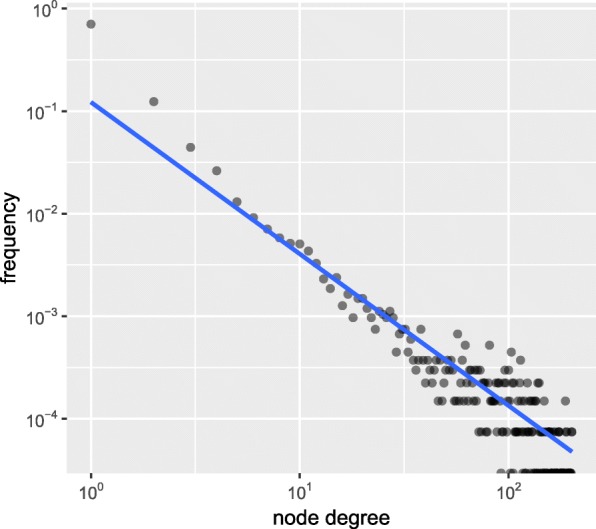
The degree distribution of the co-methylation network. The tendency of the degree on the log-log plot fits with power law, and the degree exponent of this network is close to 2, thus the power-law degree distribution conforms to scale-free network topology

The methylation data of 13,415 sites under 32 conditions was used to construct the co-methylation network. Since the location of these sites are known, the genes where these sites correspond to (Entrez Gene ID & Gene Symbol) were labeled. A total of 52 sites were dropped due to the absence of relevant gene annotation, and the remaining sites were kept for network construction. The site pair was defined as the co-methylation site pair only if its scc is ranked in the highest or lowest 10% and its adjusted *p*-value is lower than 0.05. According to the above strategy, the adjacency matrix was constructed to obtain the linkage between site pairs. The function in package igraph transformed the format of the matrix to the igraph format. A network consisting of 18,477 edges and 13,363 nodes was constructed. The constructed network with the most significant functions of four main modules in Cytoscape is shown in Fig. 2. We observed that majority sites are clustered together in a huge group, where several modules can be identified. Moreover, we obtained small clusters ranging between 2 and 9 sites. To have a better understanding of the network, we looked at the degree distribution (see Fig. 3), which unveiled that this co-methylation is a typical scale-free network. The scale-free network tallies with most biology networks for its robustness against disruptions. In the scale-free network, highly connected hubs, making up a relatively small number of nodes, will mainly are pivotal in determining the property of the network. The log-log plot gives an almost linear trend, with the degree exponents to be around 2.

Additionally, it should be of great interests to compare the constructed co-methylation network to the co-expression network. For this purpose, we downloaded the human co-expression data (Coexpression version: Has-u.c2–0) from COXPRESdb [27], and built the gene co-expression network with cut-off threshold 0.8, i.e., if the Pearson correlation value between two genes is more than 0.8, the gene pair are considered co-expressed. Meanwhile, a gene-gene co-methylation network was converted from the site-site co-methylation network constructed previously. If two sites are co-methylated, their hosting genes are considered co-methylated as well. The gene-gene co-methylation network was then compared to the gene-gene co-expression network. However, we failed to observe strong topological correlation between the co-expression and co-methylation networks. Although is still positive, the Pearson correlation of their adjacency matrices is only 2.2E-4, which suggests the epitranscriptome regulatory impact of transcriptional expression may be relatively weak at global level.

### Hub-based method

The annotation of methylation sites relies on the functional enrichment in the hosting genes of their neighbor sites according to the guilt-by-association principle. Because the functional information of individual RNA methylation site is unavailable in existing database, we consider a soft benchmark by assuming that the functions of a sites are similar to that of its hosting genes. In the network, 1899 (14.2%) sites with connections to more than 3 immediate neighbors are defined as hub sites. To evaluate the accuracy of the prediction, we also annotated the predicted functions with the known GO terms of their corresponding gene. Thus, the enriched GO BP terms of genes where these hub sites correspond to were annotated with the Entrez ID using packages GO.db and AnnotationDbi. The corresponding genes of 1780 hub sites were annotated with GO BP terms. We also annotated all the neighboring sites of each hub site with GO BP terms. Both the annotated terms were reduced to GO Slim BP terms, and the term GO:0008150 (biological process) was excluded in annotation results because this term almost occurs in every reduced GO Slim term. A total of 1446 sites were annotated with more than one GO slim BP term. The terms occurring as both predicted and known terms were treated as hit terms. Permutation on sites was performed to construct the random network. In the random network, the GO BP terms and GO BP slim terms of the corresponding genes of the hub-sites were the same as that in the real network, while the predicted terms by their neighbors were different. We defined recall and precision to measure the prediction performance, and two cutoff parameters PV and GN can affect the prediction performance.

**Fig. 4:**
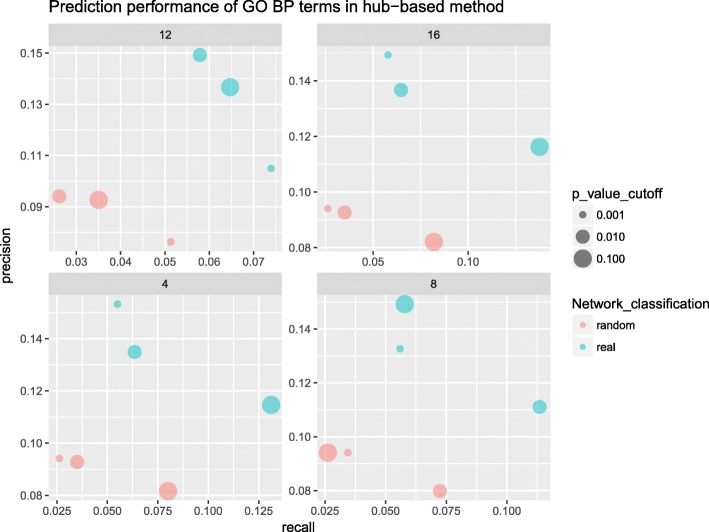
Performance of hub-based functional prediction. In the recall-precision plot, blue circles represent values under the real network, and red circles represent values under the random network. The x-axis and y-axis give the respective values of recall and precision. The number labelled as title in each facet represents each GN cutoff. The lower PV cutoff represent the smaller circle in the figure. From the figure, the values of recall and precision in the real network are much higher than the random network with the same cutoff

We showed in Fig. 4 the relationship between recall and precision values of both real and random networks under different cutoffs of PV (circle size) and GN (facet title). The points in blue are the performance values in the real network, and the points in red are the performance values in the random network. The values of recall and precision in the real network under these cutoffs are much higher than that in random network, which proves that the prediction in the real network should be of biological significance. The recall value is highest (13.8%) when the values of both GN (16) and PV (10^− 1^) cutoff are high. The precision value is highest (15.3%) when the values of both GN (4) and PV (10^− 3^) are low. The recall value is strongly affected by GN, while the precision value is affected more by PV. Therefore, the PV and GN will not be set too low or too high to get the reasonable recall and precision.

### Module-based method

**Fig. 5:**
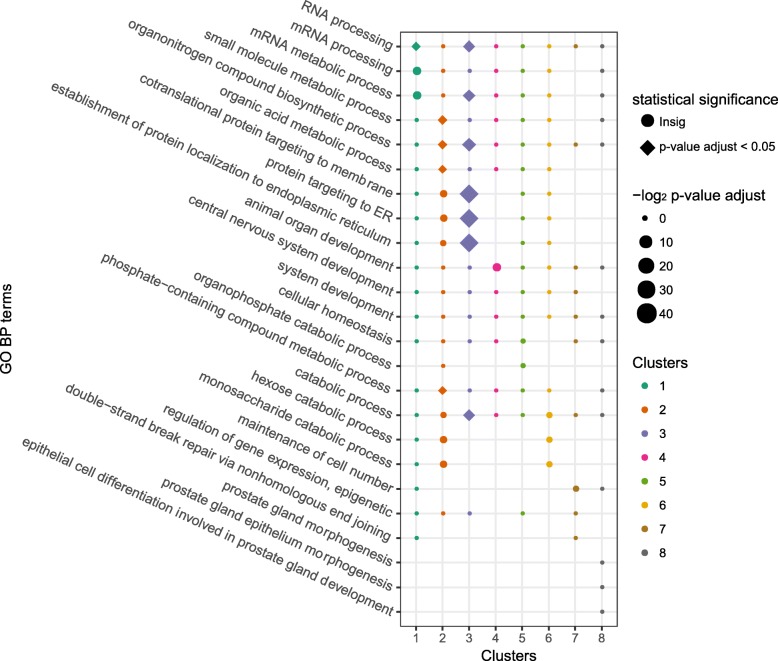
GO enrichment plot of the eight most significant modules from the module-based method. The larger the point size means the lower the *p*-value of the GO term. The shape in rectangle means the significance in statistics (p-value after BH adjustment is lower than 0.05), while the shape in circle means the insignificant term. GO BP terms such as RNA processing as well as small molecule metabolic process are statistically significant

It is highly possible that sites within a co-methylated module share similar functions. Therefore, analyzing the corresponding genes of methylation sites in the same module can help us predict the site functions in the module-based method. The igraph object after network construction was set as the input file of the clustering algorithm. After clustering the sites with MCL algorithm (inflation value set to 1.4), 76 modules (2303 sites) containing 10 or more sites were defined as modules, the enrichment analysis of GO BP terms of these modules was performed. All the modules were enriched with more than one GO BP terms whose *p*-values are lower than 0.05. After adjusting the p-value by the BH method, 8 modules were significantly (adjusted p-value < 0.05) annotated with at least one GO term. In Fig. 5**,** the enriched result of the eight modules in the module-based method is given. The enriched terms in each module are labeled using different colors and columns. The size of points in the dot plot gives an indication of the magnitude of p-values corresponding to the enriched terms. The shape of points there indicates the statistical significance of the terms. The GO BP terms in module 2 (small molecule metabolic process, organonitrogen compound biosynthetic process, etc.) and module 3 (co-translational protein targeting to membrane, protein targeting to ER, etc.) are statistically significant (shown the Fig. 5).

### Overlap of the functional enrichment

**Fig. 6:**
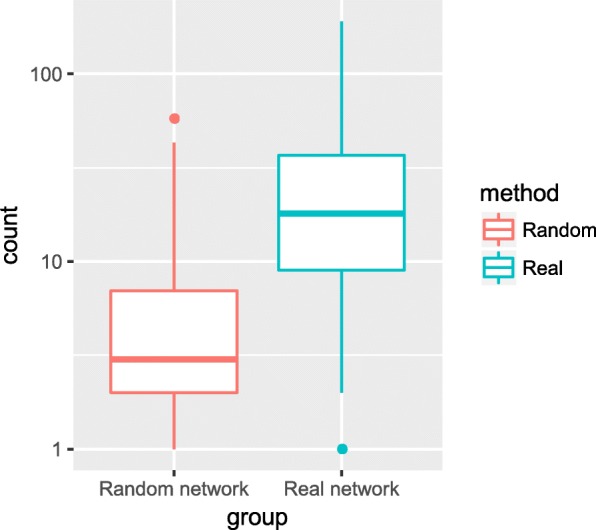
The enriched terms are more consistent in real network. The boxplot of the overlap term number in the real network and the random network at the same methylation site. The box in red represents the term count in the random network, and the box in blue represents the term count in the real network. After log_10_ transforming the term count on y-axis. It is obvious that the overlap terms are much more in the real network (mean 27) than in the random network (mean 3.2)

To evaluate the prediction accuracies of both methods, we compare the enriched functions of the same site by the two methods. Among the 2303 sites annotated by the module-based method, 1346 (58.4%) sites are annotated in hub-based method. Majority of them (1262, 93.8%) are annotated with one or more GO BP term predicted by both methods, and about 27 GO BP overlap terms occur on each site in average. We also calculate the number of overlap terms in the random network, with the findings that 61.3% (825) sites are enriched with one or more GO BP term by both methods, and 3.2 overlap terms occur on each site. Figure 6 is the boxplot which shows the count of overlap terms predicted by the hub-based method and the module-based method on each site. The number of overlapping terms of both prediction methods is higher in the real network than the random network, indicating that the predicted functions annotated by the module-based method are credible.

### Database construction

To enable the direct query of the predicted functions associated with individual m^6^A RNA methylation sites, we constructed a web site m6Acomet, which stands for functional prediction of **m**^**6**^**A** RNA methylation sites from RNA **co-met**ylation network, and is freely available: www.xjtlu.edu.cn/biologicalsciences/m6acomet. A data table, which contains the necessary information of sites, is provided, including: methylation site ID, position on chromosome, RNA strand, corresponding Gene Symbol, corresponding Gene Entrez ID, count of neighbor sites, count of corresponding genes of neighbor sites, count of GO BP terms of the hub gene, count of GO BP Slim terms of the hub gene, count of GO BP terms of predicted neighbor genes, count of GO BP Slim terms of the predicted neighbor genes, count of hit terms of the two slim term columns, and count of the GO BP terms annotated by module-based method. The detailed information, which includes the exact GO (or GO slim) terms together with the enrichment significance (*p*-value < 0.05) and its neighboring m^6^A sites in the RNA co-methylation network, will be shown if the user clicks on the relevant hyperlinks.

## Conclusions

The functional characterization of post-transcriptional modification sites by wet experiments is extremely expensive and laborious. For this reason, we propose a computational framework, for the first time, to predict the putative functions of individual RNA methylation sites from an RNA co-methylation network in large-scale. Specifically, before network construction, the methylation level on each site was estimated and normalized by DESeq2 and CQN. Several systematic biases in GC content and batch effect were adjusted. The raw predicted m^6^A sites were further filtered, and only the sites with substantial biological signals were kept for further analysis. The RNA co-methylation network was built from MeRIP-seq data profiling the transcriptome-wide RNA methylation status in 32 experimental conditions. We showed that the co-methylation network exhibit typical scale-free characteristics. The biological functions of each individual RNA methylation sites were then inferred based on the guilt-by-association principle. Two different types of algorithms were developed for functional annotation. We suppose that the regulation role of each m^6^A site should be similar to the annotation roles of its corresponding gene. For this purpose, the methylation sites with three or more edges were functionally annotated by the hub-based method. The prediction performances (recall and precision) were defined to assess the predictive efficacies of the real and random networks. The PV and GN were chosen as cutoff parameters to assess the prediction performance. The random network was constructed to compare the prediction performance with that from the real network. By taking advantage of a soft benchmark, our result showed that the recall and precision values of the real network are both higher than that of the random network with various cutoff. In other words, the prediction results in the co-methylation network suggested higher biological significance. In the module-based method, sites from largest modules (module size ≥10) clustered by MCL algorithm were annotated by GO terms. After comparing the enriched terms of the sites annotated by both methods, we found that majority of the sites share overlapping GO terms, suggesting that the functional enrichment in module-based method is reasonable. Functional annotation by different methods can extend the range of annotation terms and increase the number of predicted sites. The predictions in some cases by two methods are complementary and coherent, which reinforce the validity in prediction.

It is worthwhile mentioning that the biological function of an RNA methylation site may be different from that of its host gene. The former focused on epitranscriptome layer regulation; while the latter may be regulated through any layers of gene expression regulation, e.g., DNA methylation, post-translation modification, etc. In this work, we focused specifically on the RNA methylation profiles, which is governed by RNA epigenetics regulation and thus echo biological processes regulated at epitranscriptome layer. Although the epitranscriptome modules (or RNA co-methylation pattern) have previously been shown to demonstrate functional relevance of the RNA methylation sites [12, 13], it is, to the best of our knowledge, that we are the first to use this property for functional prediction for individual RNA methylation sites.

The annotation result of the human m^6^A sites in our project are presented in an online database m6Acomet. It supports the query with respect to a biological function or a number of co-methylated RNA methylation sites, and may serve as a source of reference for further biological research.

However, the project still has a few limitations. For example, the annotation rates for all the filtered methylation sites in both methods are not satisfactory. The criteria for the construction of the co-methylation network may be too stringent and could be further optimized; more data sources such as protein-protein interaction, pathways, can be integrated with the RNA co-methylation network for a more accurate functional annotation.

## Methods

### mi-CLIP and m^6^A-CLIP supported m^6^A sites

A total of 69,446 human m^6^A sites reported by six mi-CLIP and m^6^A-CLIP experiments, which profiles the m^6^A epitranscriptome at base-resolution, were obtained from the WHISTLE project [20, 21, 28–30]. The m^6^A sites were labeled positive and retained for the following analysis if it embodies the DRACH consensus motifs of m^6^A modification and were supported by at least two out of the total six samples.

### MeRIP-Seq data for quantifying the RNA methylation level

The mi-CLIP and m^6^A-CLIP report only the location of the methylation site, but do not provide direct quantification of the methylation level of these sites. The information of the methylation level was obtained from MeRIP-Seq data. Specifically, 32 samples in 10 publicly human m^6^A MeRIP-Seq data sets from published studies were obtained from public database. All these samples contain both IP and Input data, and most of them were selected from the epitranscriptome database MeT-DB [31], with which it is now possible to construct the RNA co-methylation network. The biological replicates under the same cell line and from the same laboratory were merged, and the methylation level of the combined sample is essentially the average of all the biological replicates. Moreover, several outlier samples such as the sample from HepG2 cell line with heat shock treatment were dropped before the construction of the network due to low quality. Table 1 summarizes the data sets used in this project. All the original data were downloaded in SRA format from Gene Expression Omnibus, and the reads were aligned to human reference genome (hg19/GRCh37) with aligner Tophat2 [32].

**Table 1:** Datasets used in the study

ID	GEO accession	Cell line	Treatment	Source
1–4	SRR456542-SRR456549, SRR456551-SRR456557	HepG2	UV, HGF, IFN, UT	[39]
5–6	SRR903368-SRR903379	U2OS	CTL, DAA	[40]
7–10	SRR847358-SRR847377	HeLa	Ctrl, METTL14-, METTL3-, WTAP-	[41]
11–12	SRR1182582-SRR1182590	ES/NPC	hNPC, hESC	[42]
13–18	SRR1182591-SRR1182596, SRR494613-SRR494618, SRR5080301-SRR50312	Hek293T, Hek293A	Ctrl, WTAP-, METTL3-, METTL16-	
19–21	SRR1182597-SRR1182602	OKMS	D0, D5_WITH_DOX, D5_WO_DOX	
22–26	SRR1182603-SRR1182630	A549	Ctrl, METTL14-, METTL3-, WTAP-, KIAA1429-	
27–28	SRR3066062-SRR3066069	AML	Ctrl, FTO+	[43]
29–30	SRR5239086-SRR5239109	AML2	Ctrl, METTL3-	[44]
31–32	SRR1035213-SRR1035224	ESC	T0, T48	[45]

### Processing the methylation data

The R package DESeq2 [22] was applied to estimate the methylation level at each m^6^A site. All the samples were labeled with conditions (IP and Input) and sequence types (Single-end and Paired-end), and the reads count matrix was generated by counting the reads which share overlaps with bins. These bins are 101 bp long with each methylation site located at the center. The methylation level was then quantified by calculating the fold enrichment of reads in the IP sample compared with the input control sample with DESeq2, which uses shrinkage estimation and considers the over-dispersion of reads. This step produces the quantification result of the logarithmic fold change indicating the methylation level of each site. However, we found that conditions from the same laboratory cell line could not be classified into the same group by hierarchical clustering. We suppose that the GC content, which is the common systematic bias when dealing with RNA-seq data, could be further reduced. Therefore, the output of DESeq2 was first normalized by package CQN [33] to reduce the GC bias. After this additional bias correction, the estimated methylation level after the normalization by CQN does not show any GC content bias, and we can see that the conditions from the same cell line could be clustered together.

### Site filtering

The methylation sites need to be filtered due to low estimation accuracy on part of the raw sites. These sites were filtered by the following steps:

i. The methylation level will be masked NA if the expression value is lower than 8, or the count number on (IP + Input) samples of the same site is lower than 50. Throughout all the 32 conditions, sites should be dropped if too many missing values (NA count > 15) occur.
ii. Filtering the neighboring sites helps reduce the influence of replication on functional prediction. If the distance between two sites is too small, e.g., less than 50 bp, due to limited resolution of the m6A-seq technology, it is highly possible that they are located on the same gene and be annotated with the same function. We ought to keep one of them for further annotation. The Spearman correlation between two methylation sites which are located closer than 101 bp is calculated. If the correlation between them is above 0.8, they may be in fact corresponding to the same m^6^A site but incorrectly captured twice due to limited resolution of the m6A-seq technology. In that case, the site with lower methylation level will be dropped.
iii. Since a larger variance among different conditions indicates more obvious functions, sites with median absolute deviation of methylation level value across different conditions higher than 0.4 will be retained.

After site filtering, the site number was reduced from 69,446 to 13,415, following which quantile normalization was performed to remove potential batch effect.

### Construction of the RNA co-methylation network

The RNA methylation data which contains the methylation level of 13,415 sites over 32 conditions was used to construct the RNA co-methylation network. In the beginning, the Spearman correlation between each site pair was computed. Fisher’s asymptotic distribution was applied to estimate the *p*-value of each Spearman correlation coefficient (scc), and the p-value of each site pair was adjusted with Bonferroni method. The scc *p*-value for each gene pair with Fisher’s asymptotic test was implemented with function corPvalueFisher in package WGCNA [34]. The *p*-values were adjusted with Bonferroni method with function mt.rawp2adjp in package multtest. Site pairs with high spearman correlation (correlation value ranked in the top or bottom 10%) and low p-value (lower than 0.05) for their methylation levels are regarded to be significant co-methylation pair. The adjacency matrix was then built to denote the correlation in methylation level between each pair of sites. To build the network, function graph.adjacency in package igraph was applied to create the graphs file, and the degree distribution of this co-methylation network can be visualized. The power-law degree distribution indicates that our co-methylation network is a typical scale-free network [35], which means that the majority of the nodes in the network are connected with few other nodes, while the minority of the hub nodes are connected with plenty of nodes. Moreover, the network topological property will be visualized and analyzed with the professional network investigation software such as Cytoscape [36]. The function exportNetworkToCytoscape in package WGCNA can export the file from the adjacency matrix for visualization in Cytoscape [36].

The function of each m^6^A site was annotated with two different algorithms: hub-based method and module-based method.

### The hub-based method

From the degree distribution of our co-methylation network, it is observed that the minority of hub sites are related to large number of methylation sites. Since these hub sites play a significant role in the whole network, it would be of interest to investigate their functions in human biological process. The function neighbors in package igraph [37] helped us find the neighboring sites of each m^6^A site.

In hub-based method, the function of the hub methylation site is determined by the enrichment result of its neighbor sites, and only the sites with more than three immediate neighbor sites are treated as the hub sites. A total of 1889 hub sites remained if only those with more than 3 edges are considered. Before annotating the function, we assume that the functions of the methylation sites are the same as the ones in their corresponding genes. The Entrez Gene ID and Gene Symbol of the gene corresponding to each hub site and their neighboring sites were labeled for annotation. Therefore, the enriched GO BP terms of the neighboring sites on the genes may convey the function of the hub site. In addition, the functions of the gene where the hub-site located can reflect the role of the hub site. We performed the GO BP enrichment analysis on the corresponding gene of the hub site and the corresponding genes of its neighboring sites. The GO slim terms, which are the subset of GO term, were applied to reduce the GO enriched terms. This generic subset is used as the scope of GO Slim. Since the term GO:0008150 (biological process) is too general, it was removed from the analysis as well. The consistent terms between GO Slim BP terms of the hub gene, where each hub site located, and GO Slim BP terms of the neighbor genes, where the neighbor sites of the same hub site located, were treated as the reliably predicted functions of each hub site. To evaluate the prediction performance of the predicted GO terms, the functional enrichment *p*-value of the slim term (PV) and the number of the enriched slim terms of neighbor sites (GN) are calculated and set as the cutoff parameters. The recall and precision of the prediction performance are defined as:

$$ \mathrm{Recall}=\frac{\sum \mathrm{Both}\ \mathrm{known}\&\mathrm{predicted}\ \mathrm{GO}\ \mathrm{term}\ \mathrm{number}}{\sum \mathrm{Known}\ \mathrm{GO}\ \mathrm{term}\ \mathrm{number}} $$

$$ \mathrm{Precision}=\frac{\sum \mathrm{Both}\ \mathrm{known}\&\mathrm{predicted}\ \mathrm{GO}\ \mathrm{term}\ \mathrm{number}}{\sum \mathrm{Predicted}\ \mathrm{GO}\ \mathrm{term}\ \mathrm{number}} $$

GO terms of the hub site, the corresponding neighbors, and their overlap on each PV and GN cutoff were generated separately by previous data. The values of recall and precision for each cutoff were calculated to evaluate the prediction performance.

### Permutation on the network

To assess the efficacies of the predicted GO terms, permutation on sites was performed to rebuild the random network. If both the recall and precision values of real network are much higher than that of the random network, the predicted functions in the functional network should be biologically significant. The functions rewire and keeping_degseq in the igraph package were used to randomly rewire the edges without creating multiple edges, keeping the degree distribution of the raw graph unchanged without loop edges. The rewiring algorithm substitutes two arbitrary edges in each step ((a, b) and (c, d)) with the edges which are not existed in the raw graph as ((a, d) and (c, b)). The exchanging steps were repeated 100 times for the original graph. After the permutation, the number of neighbors of the same site does not change. This is similarly carried out on the random network as well. Since it is highly possible that the neighboring sites of the same hub site correspond to the same gene in the real network, this might result in a lower overall *p*-value of terms annotated in the random network. We constrained the neighbor gene number of the same hub site in the random network as that in the real network. The GO and GO slim terms enriched in permutation network together with other parameters were used to predict the function of methylation sites. The results of the random network were compared to that of the real network in terms of the performance of prediction.

### The module-based method

Another way to predict the site function is to investigate the modules in the network. It is common that sites among the same co-methylated module share similar functions. In the module-based method, the Markov Cluster (MCL) algorithm [38] is chosen as the clustering algorithm in grouping methylation sites. MCL is a scalable cluster algorithm, which is based on the stochastic flow in graphs to identify modules with random walk. We transformed the co-methylation network into the MCL input format, which contained the information of two nodes (sites) and the edge weight between nodes. With the inflation value set to 1.4 by default, the modules containing more than 9 sites were identified to be significant modules. These clustered sites will then be annotated according to the Gene Ontology of their hosting genes. The terms of the same site annotated by module-based method and hub-based method were then compared to test the annotation accuracy.

## Additional file

Additional file 1: **Figure S1**. The histogram of odds ratios between adjacency matrix built by all the 32 samples and with one sample removed. There are no obvious outliers corresponding to samples that will induce substantial topological changes to the co-methylation network. **Figure S2**. Topological changes induced to the co-methylation network. The topological changes induced to the co-methylation network by samples with enzyme permutation are not bigger than the other samples. (ZIP 277 kb)

## Abbreviations

BP: Biological process
CC: Cellular compartment
GO: Gene ontology
m^6^A: N^6^-methyladenosine
MeRIP-Seq: Methylated RNA immunoprecipitation sequencing
MF: Molecular function
