# Supplementary material for: m6Acomet: large-scale functional prediction of individual m6A RNA methylation sites from an RNA co-methylation network
Source: BMC Bioinformatics. 2019 May 2;20:223. doi: 10.1186/s12859-019-2840-3 (PMC6498663; doi:10.1186/s12859-019-2840-3)
Supplement: Supplementary file 1 — Figure S1. The histogram of odds ratios between adjacency matrix built by all the 32 samples and with one sample removed. There are no obvious outliers corresponding to samples that will induce substantial topological changes to the co-methylation network. Figure S2. Topological changes induced to the co-methylation network. The topological changes induced to the co-methylation network by samples with enzyme permutation are not bigger than the other samples. (ZIP 277 kb) [file 12859_2019_2840_MOESM1_ESM.zip › supplement.docx]

**Supplementary Materials for**

m6Acomet: large-scale functional prediction of individual m6A RNA methylation sites from an RNA co-methylation network

**S1. Outlier detection of the samples**

To test for sample independence, we followed these steps:

- Remove each individual sample from the original 32 samples in the methylation-level matrix.
- Build the adjacency matrix using the same procedures described previously in the manuscript.
- Since the matrix generated from previous section is filled with “0” and “1” and the dimensions of two matrices are the same as well, we can compare their topological similar by calculating the odds ratio (OR) between the adjacency matrix of original and new one generated with one sample removed.

The histogram of odds ratios between adjacency matrices built by all the 32 samples and with one sample removed is shown in **Figure S1**. All the OR values are very large, ranging between 2400 and 2700, which means that the topological connections are 2000 times more likely to be consistent with each other compared with the random permutation. There are no obvious outliers corresponding to samples that will induce substantial topological changes to the co-methylation network

**
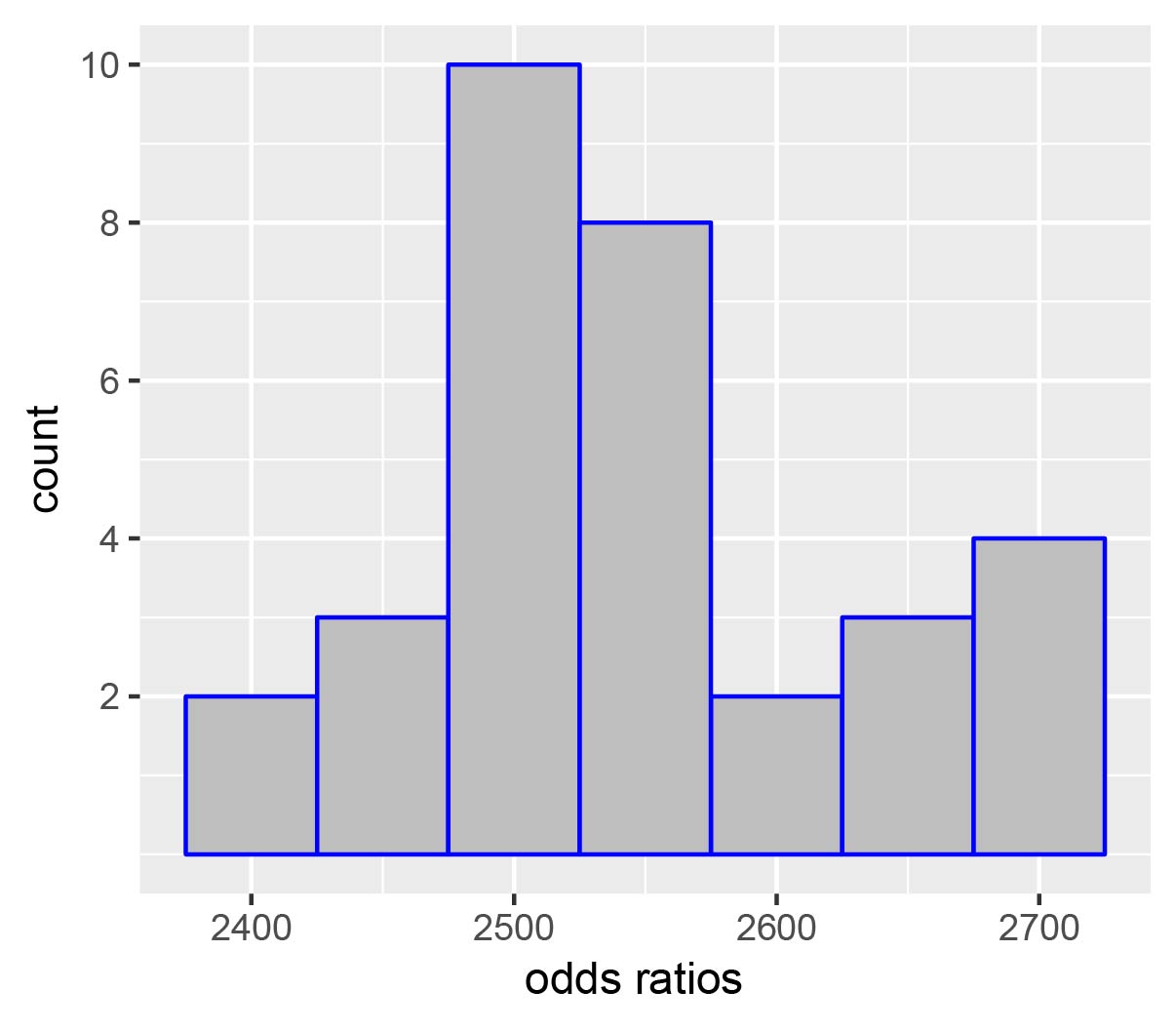
**

**Figure S1. The histogram of odds ratios between adjacency matrix built by all the 32 samples and with one sample removed.** There are no obvious outliers corresponding to samples that will induce substantial topological changes to the co-methylation network.

**S2. The enzyme perturbation samples**

To prove whether samples with m6A enzymes (METTL3, METTL14, FTO etc.) perturbation would induce bias to the co-methylation network. We followed similar procedure described previously. As shown in **Figure S2**, the topological changes induced by samples with enzyme permutation are actually slightly smaller than the other samples, as indicated by higher consistency between the adjacency matrices. Given that the number of MeRIP-seq samples is very limited, we believe it is better to keep all samples for the following analysis.

**
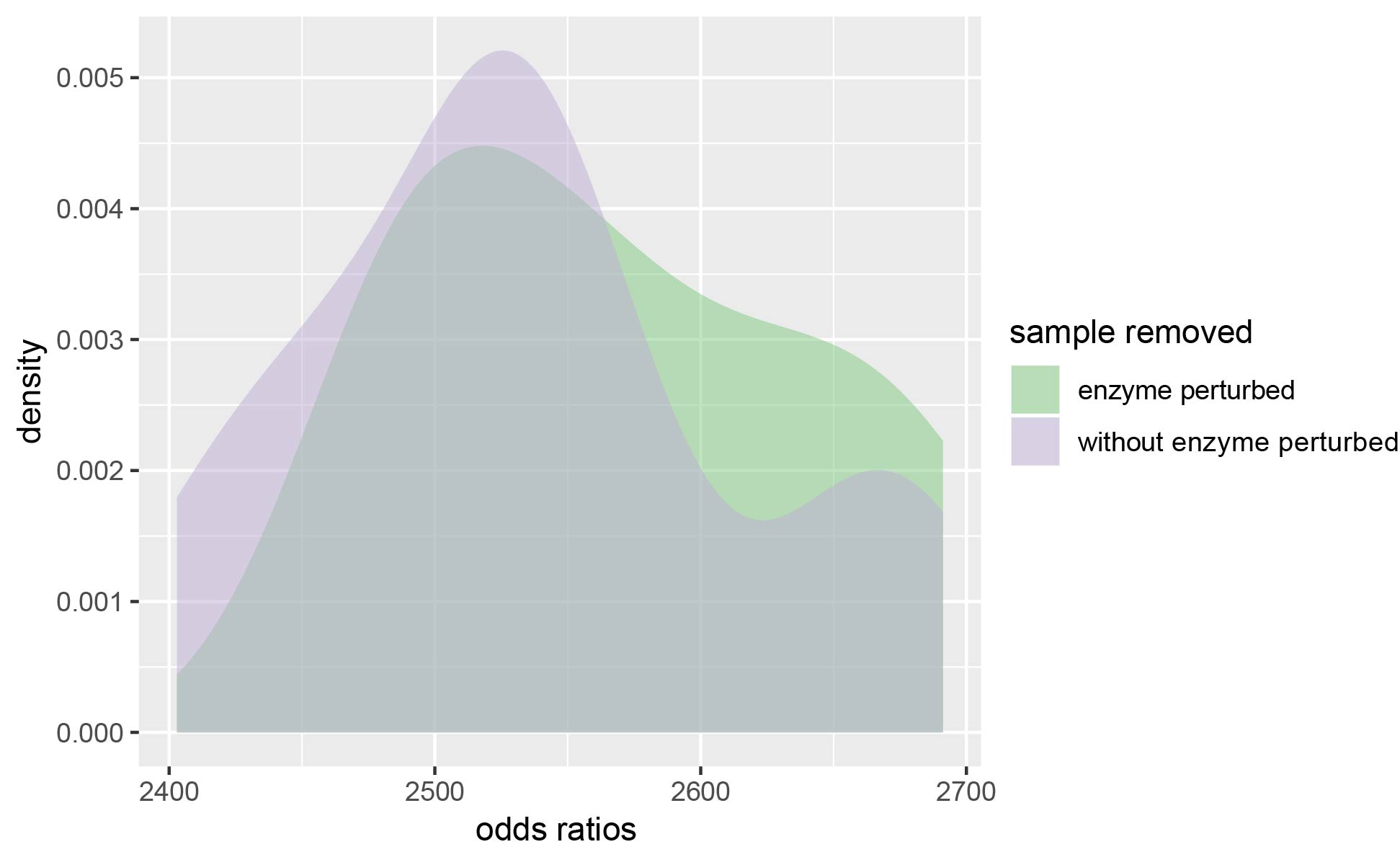
**

**Figure S2. Topological changes induced to the co-methylation network.** The topological changes induced to the co-methylation network by samples with enzyme permutation are not bigger than the other samples.
