# Supplementary figures and images for: m6Acomet: large-scale functional prediction of individual m6A RNA methylation sites from an RNA co-methylation network
Source: BMC Bioinformatics. 2019 May 2;20:223. doi: 10.1186/s12859-019-2840-3 (PMC6498663; doi:10.1186/s12859-019-2840-3)

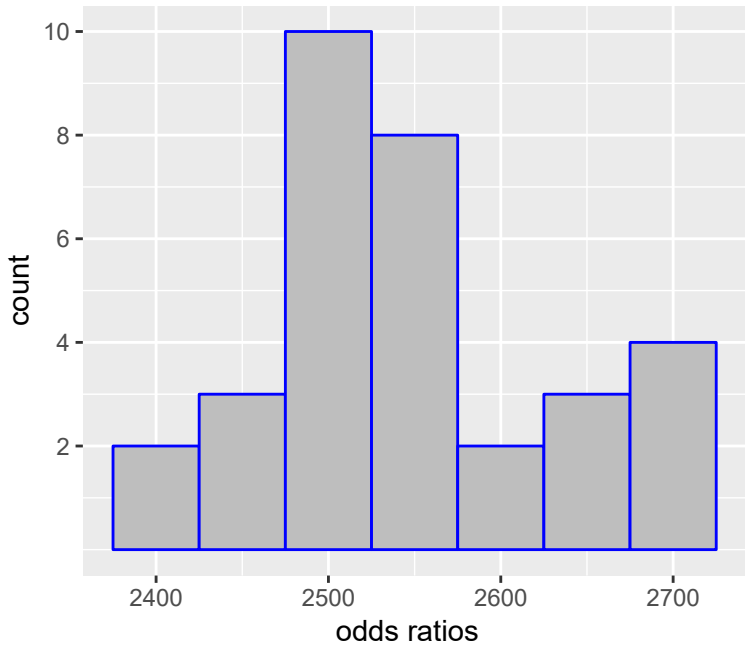

Supplement: Supplementary file 1 — Figure S1. The histogram of odds ratios between adjacency matrix built by all the 32 samples and with one sample removed. There are no obvious outliers corresponding to samples that will induce substantial topological changes to the co-methylation network. Figure S2. Topological changes induced to the co-methylation network. The topological changes induced to the co-methylation network by samples with enzyme permutation are not bigger than the other samples. (ZIP 277 kb) [file 12859_2019_2840_MOESM1_ESM.zip › Figure S1_sample_histogram.pdf]

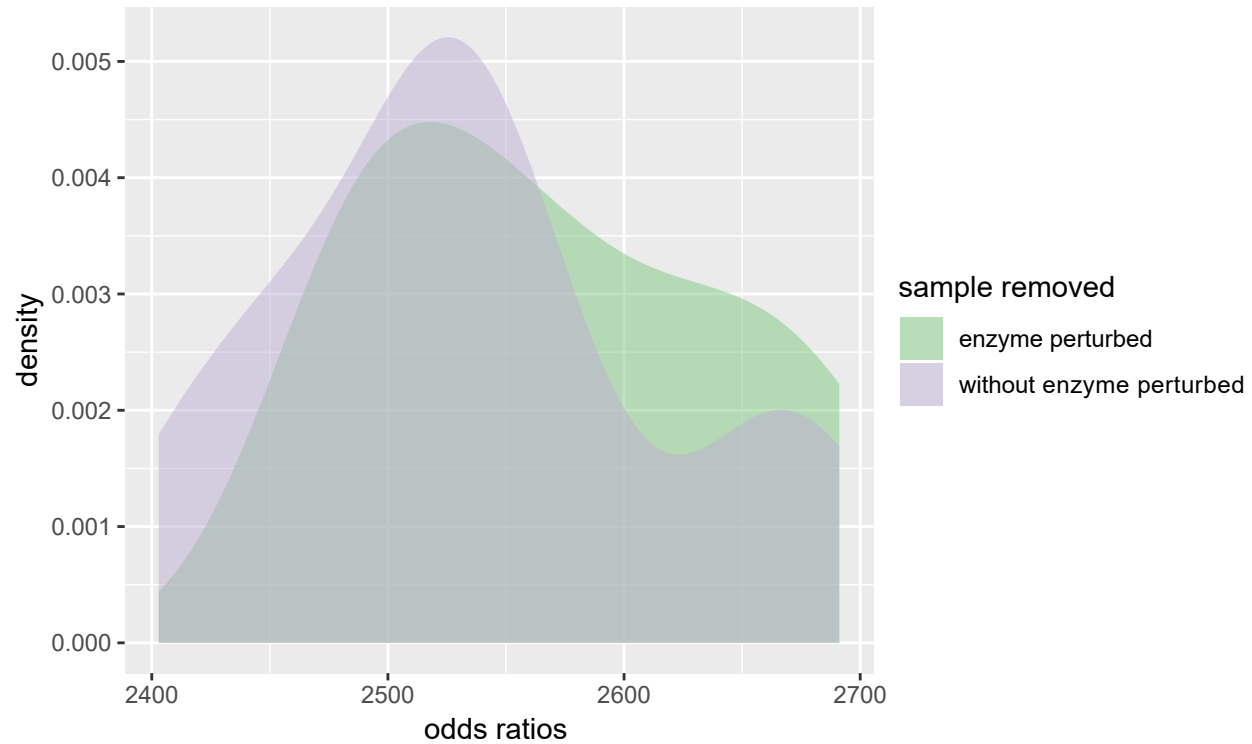

Supplement: Supplementary file 1 — Figure S1. The histogram of odds ratios between adjacency matrix built by all the 32 samples and with one sample removed. There are no obvious outliers corresponding to samples that will induce substantial topological changes to the co-methylation network. Figure S2. Topological changes induced to the co-methylation network. The topological changes induced to the co-methylation network by samples with enzyme permutation are not bigger than the other samples. (ZIP 277 kb) [file 12859_2019_2840_MOESM1_ESM.zip › Figure S2_two_kinds_of_samples_density.pdf]
